# Association of Rurality With Availability of Youth Mental Health Facilities With Suicide Prevention Services in the US

**DOI:** 10.1001/jamanetworkopen.2020.21471

**Published:** 2020-10-22

**Authors:** Janessa M. Graves, Demetrius A. Abshire, Jessica L. Mackelprang, Solmaz Amiri, Ashley Beck

**Affiliations:** 1 College of Nursing, Washington State University, Spokane; 2 College of Nursing, University of South Carolina, Columbia; 3 School of Health Sciences, Swinburne University of Technology, Melbourne, Victoria, Australia; 4 Elson S. Floyd College of Medicine, Washington State University, Spokane; 5 Community Health Department, Spokane Regional Health District, Spokane, Washington

## Abstract

This cross-sectional study uses 2017 to 2019 data from the US National Mental Health Services Survey and examines whether there are disparities in the availability of youth-serving mental health facilities associated with suicide risk in rural vs urban youth.

## Introduction

Between 2014 and 2017, the incidence of suicide increased 10% annually among US youth aged 15 to 19 years.^1^ Rural youth have a greater incidence of suicide than urban youth,^2^ a geographic disparity that has increased over time.^2,3^ From 2010 to 2018, the incidence of suicide among youth aged 10 to 19 years increased 1.5 times faster in rural areas compared with urban areas.^4^

Low health care practitioner density is associated with higher suicide rates,^5^ and youth in rural counties have access to fewer mental health services than those in urban and suburban counties.^6^ Previous research on rural and urban youth mental health services did not reflect the spectrum of rurality and did not examine the availability of suicide prevention services.^6^ The current study used 2 taxonomies of rurality to document the distribution of US mental health facilities that serve youth and offer suicide prevention services.

## Methods

In this cross-sectional study, data on 9475 US mental health treatment facilities were obtained from the Substance Abuse and Mental Health Services Administration Behavioral Health Treatment Services Locator (downloaded May 8, 2019); facility data were derived from the National Mental Health Services Survey (2017 to 2019). The publicly available data used in this study consisted of facility information and do not meet the definition of human participants research, according to the Federal Policy for the Protection of Human Subjects; therefore, institutional review board and ethics committee approval was not necessary. Facilities included outpatient, inpatient, and residential treatment facilities in all states and the District of Columbia. Facility rurality was based on 2 rural-urban taxonomies. Rurality was classified using rural-urban commuting area codes according to ZIP Code Tabulation Areas (ZCTAs; ie, metropolitan, micropolitan, small town, rural areas). Rurality was also classified by county using 2013 rural-urban continuum codes (RUCCs), which range from 1 (most urban) to 9 (most rural). Youth-serving facilities were defined in the data source as those providing services to children aged 12 years or younger or to youth with serious emotional disturbance or offering therapeutic foster care. Facilities were coded as offering suicide prevention services, which included assessment and management of suicide risk and referrals to followup care, as appropriate. This study followed the Strengthening the Reporting of Observational Studies in Epidemiology (STROBE) reporting guideline.

Using Stata/MP statistical software version 15.1 (StataCorp), we assessed the proportion of geographic areas with at least 1 youth-serving facility and with at least 1 youth-serving facility with suicide prevention services across both rural-urban taxonomies using the nptrend (nonparametric test for trend) function. The significance threshold was set at *P* < .05, and testing was 2-sided using the nonparametric test for trend across ordered groups. Statistical analysis was performed from February to April 2020.

## Results

**Figure 1.**
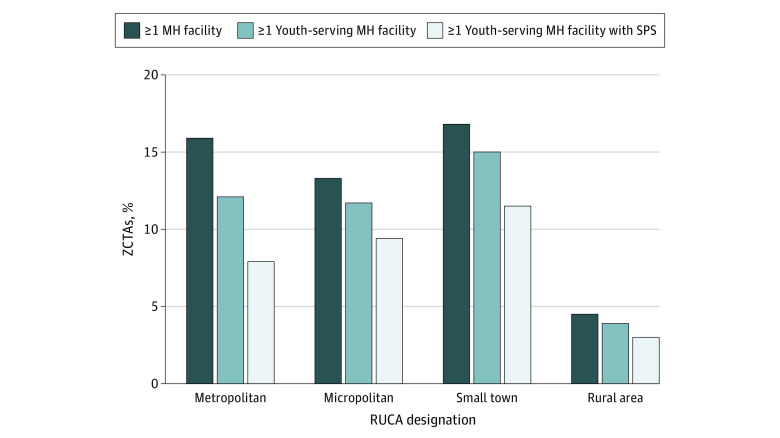
Percentage of US ZIP Code Tabulation Areas (ZCTAs) With Each Type of Mental Health (MH) Facility, Across Rurality Nonparametric tests for trend indicated significant variation in the distribution of facilities across ordered levels of rurality for all facility types (*P* < .001). RUCA indicates rural-urban commuting area; and SPS, suicide prevention services.

Across 41 083 ZCTAs, 13.7% (5637 ZCTAs) had at least 1 youth-serving mental health facility, with 3.9% (265 ZCTAs) of rural ZCTAs having a youth-serving facility, compared with 12.1% (2968 ZCTAs) of metropolitan, 11.7% (666 ZCTAs) of micropolitan, and 15.0% (615 ZCTAs) of small town ZCTAs (nonparametric test for trend, *P* < .001) (Figure 1). Similarly, 3.0% (202 ZCTAs) of rural ZCTAs had a youth-serving facility with suicide prevention services, vs 7.9% (1936 ZCTAs) of metropolitan, 9.4% (535 ZCTAs) of micropolitan, and 11.5% (472 ZCTAs) of small town ZCTAs (nonparametric test for trend, *P* < .001).

**Figure 2.**
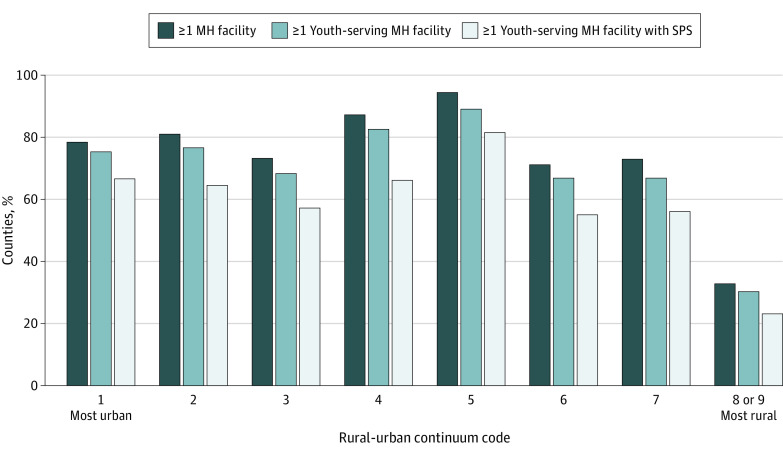
Percentage of US Counties With Each Type of Mental Health (MH) Facility, Across Rurality Nonparametric tests for trend indicated significant variation in the distribution of facilities across ordered levels of rurality for all facility types (*P* < .001). SPS indicates suicide prevention services.

Among 3223 counties, 63.7% (2053 counties) had at least 1 youth-serving mental health facility. A smaller proportion of highly rural counties (RUCC 8/9) had at least 1 youth-serving mental health facility (29.8%; 192 counties) compared with more urban counties (Figure 2). Proportionally more youth-serving mental health facilities with suicide prevention services were in counties in the middle of the rural-urban continuum (RUCC 5) compared with more urban (RUCC 1-4) or more rural (RUCC 6-9) counties.

## Discussion

The distribution of mental health facilities was comparable across US metropolitan, micropolitan, and small town areas. However, highly rural areas had fewer facilities in general—and fewer suicide prevention services in particular—compared with more urban areas.

This study had some limitations. It investigated the availability of suicide prevention services and did not examine service use. Other limitations include facility self-report of services and inability to verify the specific nature, capacity, or types of services provided.

Given the higher rates of suicide deaths among rural youth, it is imperative that the distribution of and access to mental health services correspond to community needs. Improving availability of mental health care in rural areas, alongside interventions to increase awareness, decrease stigma, and reduce access to lethal means of suicide, are critical for addressing rural-urban disparities in youth suicide.
